# Comparison of characteristics and mortality in multidrug resistant (MDR) and non-MDR tuberculosis patients in China

**DOI:** 10.1186/s12889-015-2327-8

**Published:** 2015-10-06

**Authors:** Yanni Sun, David Harley, Hassan Vally, Adrian Sleigh

**Affiliations:** National Centre for Epidemiology and Population Health, Research School of Population Health, ANU College of Medicine, Biology and Environment, The Australian National University, Canberra, 2601 Australia; School of Psychology and Public Health, La Trobe University, Melbourne, 3086 Australia

## Abstract

**Background:**

We conducted a cohort study to compare the characteristics of MDR-TB with non-MDR-TB patients and to measure long term (9-year) mortality rate and determine factors associated with death in China.

**Methods:**

We reviewed the medical records of 250 TB cases from a 2001 survey to compare 100 MDR-TB patients with 150 non-MDR-TB patients who were treated in 2001-2002. Baseline attributes extracted from the records were compared between the two cohorts and long-term mortality and risk factors were determined at nine-year follow-up in 2010.

**Results:**

Among the 234 patients successfully followed up, 63 (26.9%) were female and 171 (73.1 %) were male. MDR-TB patients had poorer socioeconomic status compared to non-MDRTB. Nine years after the diagnosis of TB, 69 or 29.5 % of the 234 patients had died (32 or 21.6 % of non-MDR-TB *versus* 37 or 43.0 % of MDR-TB) and the overall mortality rate was 39/1000 per year (PY) (27/1000 PY among non-MDR *versus* 63/1000 PY among MDR-TB). Factors associated with death included: MDR status (hazard ratio (HR): 1.86; CI: 1.09-3.13), limited education of primary school or lower (HR: 2.51; CI 1.34-4.70) and received TB treatment during the nine-year period (HR 1.82; 95 % CI 1.02-3.26).

**Conclusions:**

MDR-TB was a strong predictor for poor long-term outcome. High quality diagnosis and treatment must be ensured. Greater reimbursement or free treatment may be needed to provide access for the poor and vulnerable populations, and to increase treatment compliance.

## Background

Multidrug-resistant tuberculosis (MDR-TB) is resistant to isoniazid (H) and rifampicin (R). MDR-TB is a major barrier to TB control, especially in high burden countries such as China [1]. Despite impressive reductions in TB prevalence and mortality over the past 20 years, the World Health Organization (WHO) estimates that China has the second largest number of MDR-TB cases globally; only India has more cases [2]. The national baseline TB survey in 2007–2008 found that 8 · 3 % of cases were MDR-TB, predicting approximately 120,000 new MDR-TB cases annually [3].

MDR-TB requires prolonged treatment with costly second-line anti-TB drugs (SLD), leading to health system opportunity costs, adverse effects, and financial impacts for patients [4–6]. Patients with MDR-TB have a low treatment success rate: 48 % globally and 50 % in China [7]. Recurrence and treatment failure are more common for drug resistant than drug sensitive TB [8–10], however, little is known of the impact of multidrug resistance on long-term mortality and survival.

We conducted a follow-up study nine years after diagnosis with TB or MDR-TB in Henan Province, China to compare characteristics and mortality of MDR and non-MDR-TB patients. Our study sample was drawn from a representative drug resistance surveillance (DRS) study conducted in 2001 in Henan. We chose Henan because it had the second largest number of TB and MDR-TB cases and a high quality DRS in 2001. All culture-verified patients were identified in 2001, treated with first-line drugs (FLDs) through directly-observed treatment, short-course (DOTS) between 2001 and 2002, and surveyed in 2002 and again in 2010. We report long term outcome and hazard ratio data for death by comparing two cohorts: MDR-TB patients and non-MDR-TB patients.

## Methods

### Study design

We conducted a cohort study followed up TB and MDR-TB patients nine years after diagnosis with the disease in Henan Province, China.

### Study setting

Henan Province is in the middle of China and had a population of 99.1 million people in 2010 [11]. Although the incidence of TB in Henan (71.1 per 100,000 in 2010) is close to the Chinese average, the absolute number of TB cases reported in 2012 (68,042) was the second highest among all provinces [11]. DOTS, as recommended by WHO, has been implemented progressively in Henan Province since 1996, reaching 100 % coverage in 2005.

### Study population and field procedure

**Fig. 1:**
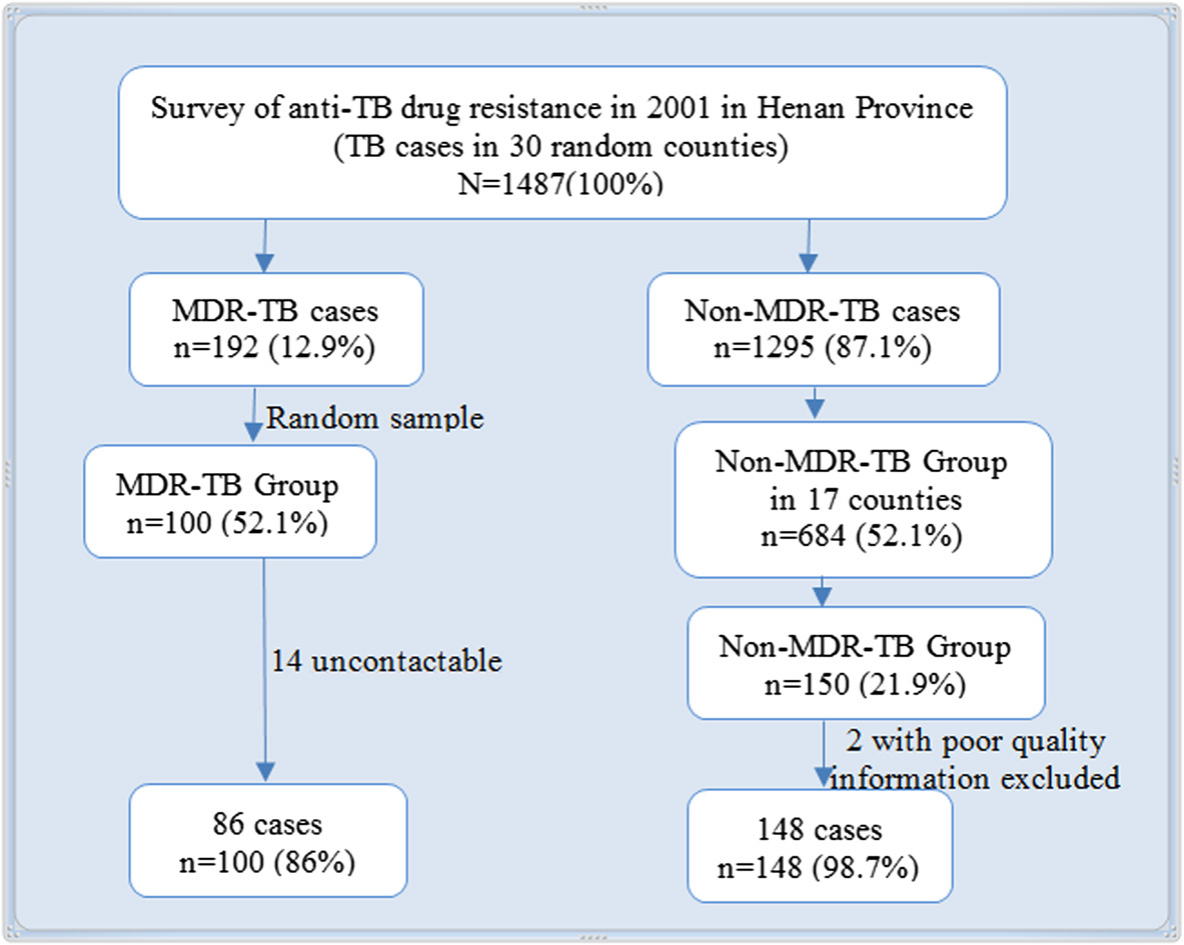
Simple random sampling processes for selecting study population in 2010

**Fig. 2:**
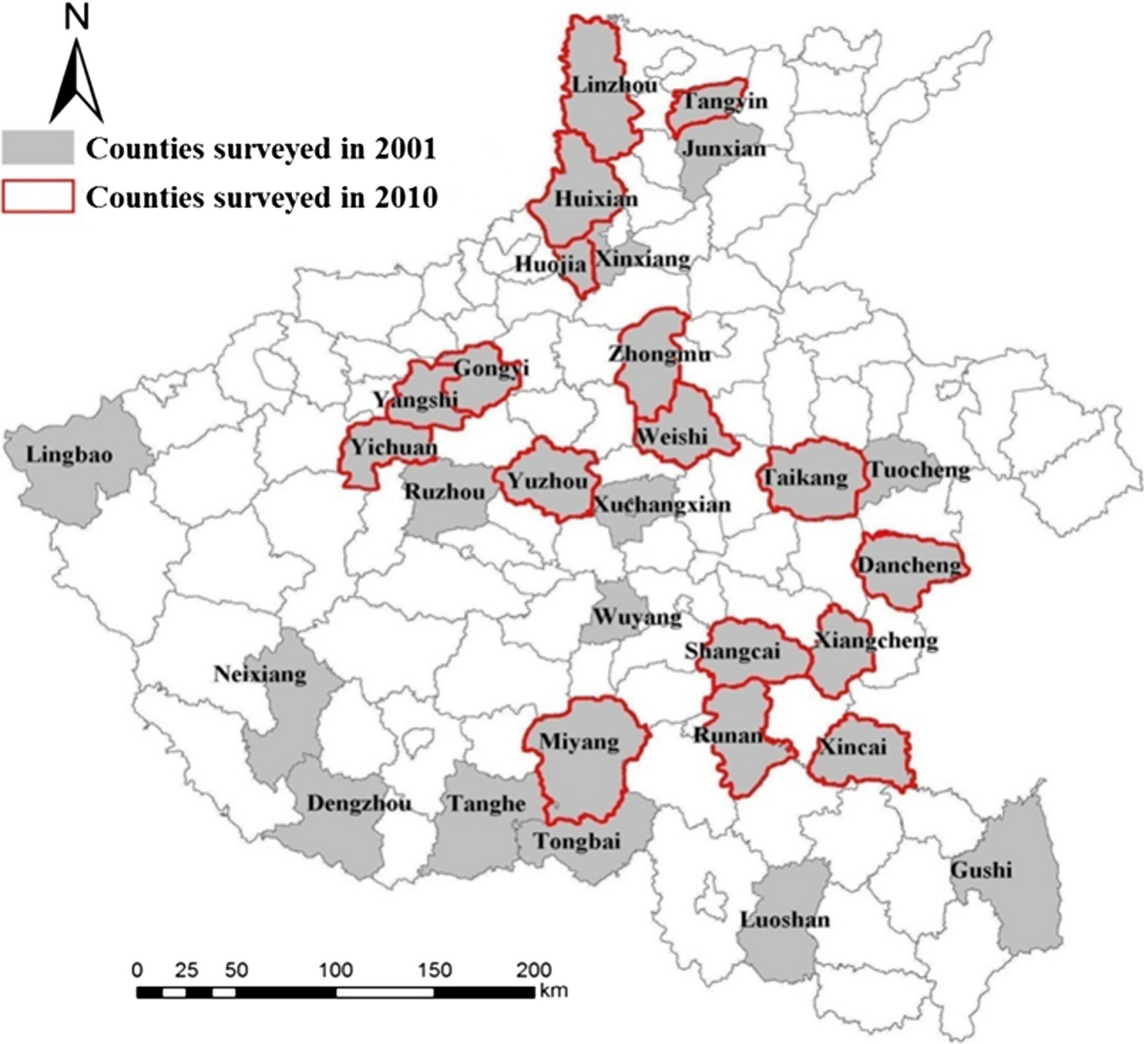
Geographical location of counties surveyed in Henan Province, 2001 & 2010

In 2001, WHO supported a DRS that was conducted in Henan Province. A total of 1854 newly registered, sputum smear-positive cases from 30 of 159 counties and districts who had been treated between July 2001 and June 2002 were selected at random from the DRS and resurveyed. The survey methods, findings, and culture results of the 1487 cases who had culture results and complete medical records are reported elsewhere [12]. Those 1487 cases included 192 culture-proven MDR-TB patients and 1295 non-MDR-TB patients [12]. All patients were eligible to be included in the follow-up study; 100 MDR-TB patients and 150 non-MDR-TB patients in 17 counties were randomly selected, based on power calculations (Figs. 1 and 2). The study was powered to detect a 2-fold increased risk of death over 10 years between the MDR and the non-MDR groups, with 2-tailed significance of 0.05 and a power of 0.8, assuming 20 % mortality in the MDR-TB group. Non-MDR patients were oversampled in a ratio of 1.5:1 to accommodate greater difficulty tracking non-MDR patients. The minimum sizes of the required samples were 112 non-MDR patients and 75 MDR patients; sample sizes were increased proportionately to 150 and 100 patients, respectively, to account for patients being lost to follow up.

In 2010, we followed up the 250 randomly-sampled patients. We extracted data on demographics, treatment history, DST results, and treatment outcomes from the DRS. All information was re-checked against patients’ medical records, with few discrepancies being found. Discrepancies that were discovered were resolved with information from county or district TB dispensaries. A questionnaire was developed based on the aims of the follow up study. Interviews were conducted at informants’ homes or at clinics from January to May 2010. Sputum status was extracted from laboratory and medical records in county or district anti-TB departments. If a patient had been reported to have died after completion of treatment in 2002, information on date and cause of death was collected. If the patient could not be interviewed because they had died, moved out of the home, or were absent from the home when the interview was conducted, we interviewed family members, neighbours, village doctors, and other village leaders to collect the required information. We were unable to contact 14 of the 100 MDR-TB patients, and we excluded two of the 150 non-MDR-TB patients due to poor data quality. The final dataset included 86 MDR-TB and 148 non-MDR-TB patients.

### Treatment regimens and outcomes

Treatment regimens followed the Chinese National Tuberculosis Control program (NTP) treatment guidelines, as recommended by WHO. Treatment of new smear-positive patients consisted of two months of H, R, pyrazinamide (Z), and ethambutol (E) followed by four months of H and R three times weekly. Patients who had previously received at least one month of TB treatment (i.e. retreatment patients) received two months of H, R, Z, S and E, followed by six months of H, R, and E three times weekly. Drug administration was observed at the health facility throughout treatment. The treatment regimens and outcomes have been presented in detail elsewhere [13]. Treatment outcomes were assessed using WHO definitions [14].^1^

### Data management and statistical analysis

All data were double entered in EpiData 3.1 (The EpiData Association, Odense, Denmark, 2008), and internal consistency was determined. Internal discrepancies were checked against the original forms by the first author. Statistical inference followed standard procedures for categorical data [15]. Pearson’s χ^2^ test or Fisher’s exact test was used for testing differences in proportions. Two-sided distribution *p*-values of <0.05 were considered significant. The overall mortality rate was calculated, as well as that specific to the two groups (MDR and non-MDR-TB), expressed in terms of cases per 1000 person-year (PY) of follow-up and at two-year intervals. The follow-up period from the date of DOTS TB treatment completion until death, transfer, completion of follow-up, or the end of the study was computed as the time elapsed since the end of TB treatment. A Cox proportional hazards regression was performed with time dependant covariates in relation to TB death; a forward inclusion approach was used. Variables significantly correlated in the unadjusted model with *p*-value <0.05 and those of epidemiological interest were included into the multivariable model. *Hazard ratios* (HR) with their 95 % confidence intervals (CI) were used to measure association. Survival curves, unadjusted and adjusted by age and other covariates over drug resistance status, were plotted. Stata software (Version 12; Statacorp) was used for data analysis and preparation of graphs.

### Ethical considerations

The original 2001 MDR-TB surveillance survey was approved by the Ethics Committee of the Department of Health of Henan Province based on Chinese national ethical regulations. The follow-up study in 2010 was approved by the Ethics Committee of the Tuberculosis Control and Prevention Institute of Henan Provincial Center for Disease Control and Prevention and by the Human Research Ethics Committee of the Australian National University on 12 November, 2009 (Protocol 2009/553, Appendix 2). Information sheets and consent forms were provided to participants, and consent was obtained from all study participants. Participation was voluntary, and confidentiality of data was maintained.

## Results

### Attributes of the study population at baseline

A total of 234 patients, 86 MDR-TB and 148 non-MDR-TB patients were identified and followed up. All were reported to have completed treatment, and none were HIV positive. Of the 86 MDR-TB and 148 non-MDR-TB patients, the sex ratios and age structures were similar (Table 1). Most (approximately 80.0 %) were married and there was no significant difference in marital status between the MDR and non-MDR-TB groups. A majority (approximately 85 %) were farmers. The groups had similar educational attainment (44 · 1 % of MDR-TB *versus* 54 · 8 % of non-MDR-TB subjects attained middle-school education or above, *p* = 0 · 19).

**Table 1:** Attributes of the study population by drug resistance status at baseline, 2001-2002^a^

Patient attributes	MDR-TB	Non-MDR-TB	*p*-value
	*n* = 86 (%)	*n* = 148 (%)	
Sex			
Male	64 (74.4)	107 (72.3)	0 · 72
Female	22 (25 · 6)	41 (27 · 7)	
Age groups (years)			
10 ~ 29	20 (23 · 3)	35 (23 · 6)	0.16
30 ~ 44	28 (32 · 6)	42 (28 · 4)	
45 ~ 59	26 (30 · 2)	33 (22 · 3)	
60~	12 (13 · 9)	38 (25 · 7)	
Marital status			
Married	70 (81 · 4)	115 (78 · 2)	0 · 84
Others^b^	16 (18 · 6)	32 (21 · 8)	
Occupation			
Farmers	75 (87 · 2)	125 (85 · 0)	0 · 29
Others^c^	11 (12 · 8)	22 (15 · 0)	
Education			
≤ Primary school	48 (55 · 9)	66 (45 · 2)	0 · 19
≥ Middle school	38 (44 · 1)	80 (54 · 8)	
Smoker	30 (34 · 9)	28 (18 · 9)	<0 · 01
Alcohol drinker	14 (16.3)	20 (13.5)	0.56
Work outside^d^ for cash after TB cured	8 (9 · 3)	3 (2 · 0)	<0 · 01
Numbers of bedrooms in patients’ house			
≥ 4	51 (62 · 2)	119 (82 · 1)	<0 · 01
≤ 3	31 (37 · 8)	26 (17 · 9)	
Annual household income (Yuan)			
≥ 10 000	38 (44 · 7)	91 (61 · 9)	0 · 01
< 10 000	47 (55 · 3)	56 (38 · 1)	
Community/government offered financial help	13 (15 · 7)	12 (8 · 8)	0 · 24
Insurance			
Insured	8 (10 · 5)	16 (11 · 0)	0 · 97
Own expense	77 (89 · 5)	130 (89 · 0)	

^a^Those with missing values were excluded from the comparisons

^b^Others include single, divorced, and widowed

^c^Others include worker, cadre, teacher, student, self-employment, unemployed, and housewife/homemaker

^d^Patient worked outside hometown for cash as a migrant worker

A greater proportion of MDR-TB patients were found to be smokers after their diagnosis (34 · 9 % *versus* 18 · 9 %; *p* < 0 · 01). There was no significant difference in alcohol consumption. More MDR-TB than non-MDR-TB patients worked following cure of their TB (9 · 3 % in MDR-TB *versus* 2 % in non-MDR-TB; *p* < 0 · 01). Patients with MDR-TB were more likely to live in houses with fewer than three bedrooms (37 · 8 % *versus* 17 · 9 %; *p* < 0.01). MDR-TB households had lower income; 55 · 3 % had annual household income less than 10,000 Chinese Yuan compared to 38 · 1 % for households with non-MDR-TB (*p* = 0 · 01). A higher percent of MDR-TB patients (15 · 7 %) than non-MDR-TB patients (8 · 8 %) received some financial support from the local community or government. Medical insurance was held by 10 · 5 % and 11 %, respectively, in the MDR-TB and non-MDR-TB groups. Uninsured patients paid for their own TB treatment.

### Mortality rates

The overall mortality rate of the followed up patients was 39/1,000 PY (11/1,000 PY among those 10–29 years of age, and 31, 52, and 75, respectively, for those aged 30–44, 45–59, and 60 years or above). The mortality was 27/1,000 PY among non-MDR-TB and 63/1,000 PY among MDR-TB patients, respectively. The highest mortality rates were found between the second and fourth year after completion of treatment (53/1,000 PY), followed by the fourth to sixth (48/1,000 PY), and the first two years (42/1,000 PY). Mortality rates for non-MDR-TB and MDR-TB patients in the first two years after the completion of treatment were 86 and 17/1,000 PY, respectively.

### Attributes of the study population with regard to long term survival by 2010

The median age of the study population was 49 years (IQR 20–84). No significant difference was observed in the ratio of males to females with TB. A statistically significant difference in mortality between MDR and non-MDR-TB patients was seen after 9 years (53.6 % *versus* 29.7 %; *p* = 0.001). A greater proportion of patients with education lower than primary school died compared to those with education level at middle school and above (71.0 % *versus* 39.4 %; *p* = 0.03). More patients who lived in houses with fewer than three bedrooms had died (34.9 % *versus* 21.1 %; *p* = 0.03). Mortality was more common in those with annual household incomes less than 10,000 Yuan (60.3 % *versus* 37.8 %; *p* = 0.002). Patients who received TB treatment during the nine-year period were more likely to die (33.9 % *versus* 16.6; *p* = 0.005) (Table 2).

**Table 2:** Attributes of the study population by long term survival to 2010^a^

Patient attributes^b^	Total	Survived	Died	*p*-value
	*n* = 234 (%)	*n* = 165 (%)	*n* = 69 (%)	
Median age (years) (IQR^c^)	49 (20–84)	46 (23–84)	58 (20–86)	0.003
Sex				
Female	63 (26.9)	49 (29.7)	14 (20.3)	
Male	171 (73.1)	116 (70 · 3)	55 (79 · 7)	0.14
Drug resistance status				
Non-MDR-TB	148 (63.3)	116 (70 · 3)	32 (46 · 4)	
MDR-TB	86 (36.7)	49 (29 · 7)	37 (53 · 6)	0.001
Education				
≥ Middle school	120 (51.3)	100 (60 · 6)	20 (29 · 0)	
≤ Primary school	114 (48.7)	65 (39 · 4)	49 (71 · 0)	<0.001
Number of bedrooms in patient’s home				
≥ 4	170 (74.9)	127 (78 · 9)	43 (65 · 1)	
≤ 3	57 (25.1)	34 (21 · 1)	23 (34 · 9)	0.03
Annual household income (Yuan)				
≥ 10,000	129 (55.6)	102 (62 · 2)	27 (39 · 7)	
< 10,000	103 (44.4)	62 (37 · 8)	41 (60 · 3)	0.002
Whether received TB treatment				
No	172 (78.5)	131 (83.4)	41 (66.1)	
Yes	47 (21.5)	26 (16.6)	21 (33.9)	0.005

^a^Those with missing values were excluded from the comparisons

^b^Variables also tested and found to have *p* value > 0.10 and not listed in the table included the following: marital status, occupation, smoker after getting TB, alcohol consumption, worked as a migrant worker after got TB cured, and not received help from social welfare and insurance

^c^Interquartile range

### Factors associated with death

**Fig. 3:**
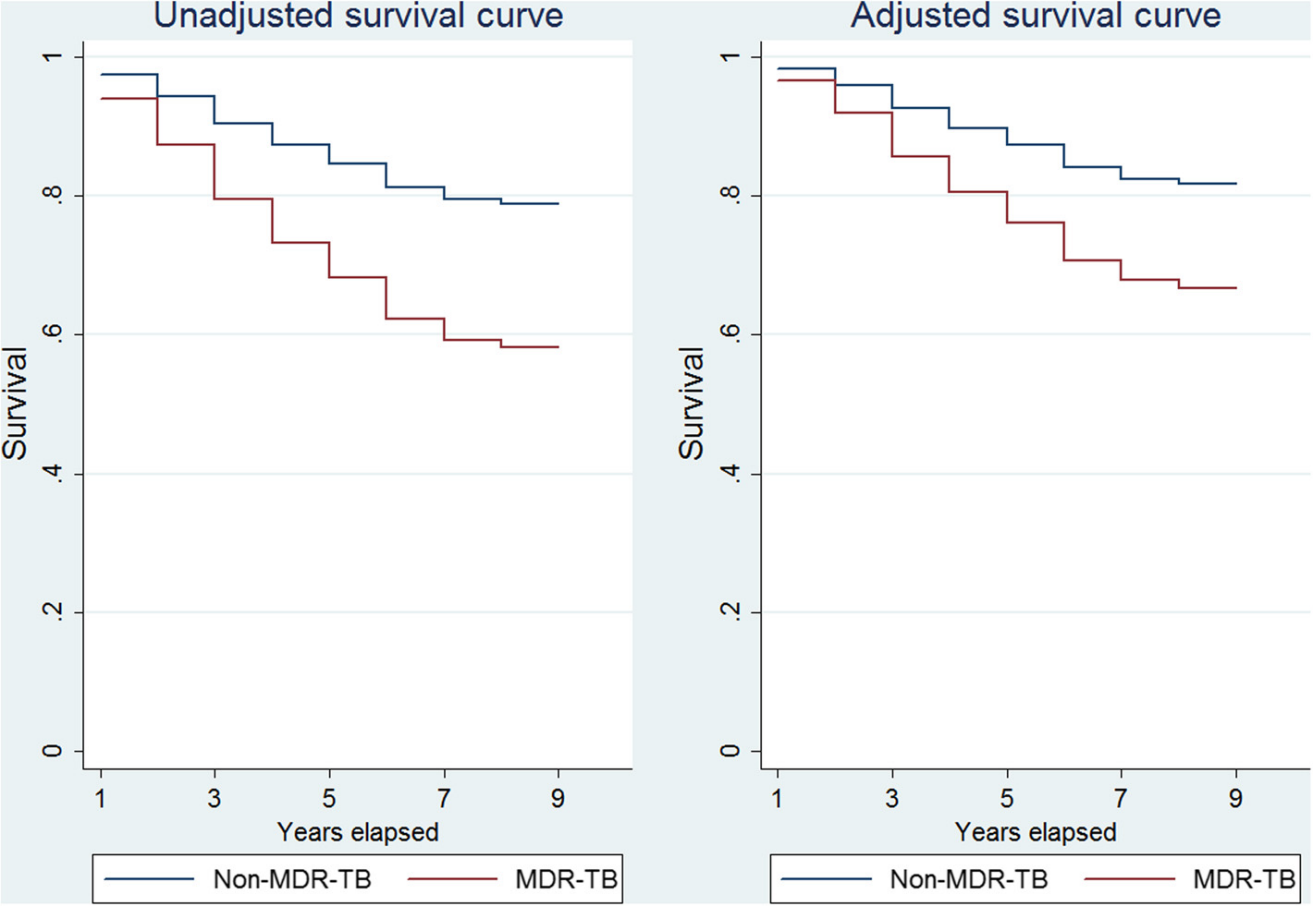
Survival analysis estimates of long-term survival based on drug resistance status

In unadjusted analysis, death was significantly associated with age greater than 45 years, MDR-TB, lower level of education, having fewer than three bedrooms in the home, having an annual household income less than 10,000 Yuan, and having received TB treatment (Table 3). In the multivariate analysis, death was significantly associated with MDR (HR 1 · 86; 95 % CI 1 · 09-3 · 13), educational attainment of primary school or lower (HR 2 · 51; 95 % CI 1 · 34-4 · 70), and having received TB treatment (HR 1.82; 95 % CI 1.02-3.26). The mean survival for MDR-TB was 6.4 years compared to 7.7 years. The survival curves are plotted by drug resistance status unadjusted and then adjusted with statistically significant covariates (Fig. 3).

**Table 3:** Factors associated with death in the study population, 2010^a^

Patient attributes	Unadjusted HR	*p*-value	Adjusted HR	*p*-value
	(95 % CI)		(95 % CI)	
Male sex	1 · 50 (0 · 83-2 · 71)	0.17	1 · 39 (0 · 76-2 · 56)	0.28
Age groups (years)				
≤ 44	1		1	
45-59	2.02 (1.04-3.92)	0.04	1.46 (0.73-2.94)	0.28
60~	2.82 (1.52-5.25)	0.001	1.80 (0.88-3.66)	0.10
Drug resistance status				
Non-MDR-TB	1.00		1.00	
MDR-TB	2 · 29 (1 · 41-3 · 71)	0.001	1 · 86 (1 · 09-3 · 13)	0.02
Education				
≥ Middle school	1.00		1.00	
≤ Primary school	3 · 18 (1 · 84-5 · 46)	<0.001	2 · 51 (1 · 34-4 · 70)	0.004
Number of bedrooms in patient’s home				
≥ 4	1.00		1.00	
≤ 3	1 · 7 (1 · 01-2 · 86)	0.04	0 · 99 (0 · 56-1 · 75)	0.97
Annual household income (Yuan)				
≥ 10,000	1.00		1.00	
< 10,000	2 · 09 (1 · 27-3 · 43)	0.004	1 · 50 (0 · 87-2 · 59)	0.14
Received TB treatment	2.12 (1.23-3.66)	0.007	1.82 (1.02-3.26)	0.04

^a^Those with missing values were excluded from the comparisons

## Discussion

This study in Henan Province, China is, to the best of our knowledge, the first comparison of long-term outcomes and mortality between MDR-TB and non-MDR-TB patients. We found that patients with MDR-TB had poorer outcomes than non-MDR-TB patients. The survival time for MDR-TB patients was shorter than for non-MDR-TB patients. We also found that drug resistance status was a very strong predictor of mortality, as was educational level attained. The provision of TB treatment was also statistically significantly associated with mortality.

Mortality in MDR-TB patients was approximately five-fold higher than among the non-MDR-TB patients in Henan, China by 2010. This MDR-TB mortality rate was higher than rates reported from studies in other regions and countries [8, 16, 17], perhaps because we followed up patients over a longer period (9-years) than others (3–6 years).

Increased age was not identified as a mortality risk factor in the final multivariable analysis in our study, unlike findings from other studies [18, 19]. Older patients may have delayed health care seeking, and diagnosis may also have been delayed due to the nature of the clinical presentation in older patients compared with younger patients. Poor immune function in older people may influence mortality [18, 20]. In our study, age was a risk factor in unadjusted analysis, but did not remain significant in multivariable analysis. This may be due to confounding of age with educational attainment, household economic status, and access to TB treatment during the nine-year follow-up period. In our study, multidrug resistance was one of the strongest risk factors for death, with a HR 1.86 times higher compared with non-MDR-TB. A similar finding was reported from a study in Estonia [19, 21].

We found a significant association between lower education and death in both unadjusted and multivariable analyses—a finding that has not been reported elsewhere [21–23]. Patients with less education may lack knowledge about health care access and the importance of compliance with treatment. Low annual household income was also found to be associated with death. Poverty, low household economic status, and lack of health insurance have all been reported as risk factors in previous studies [23].

Another key finding from our study was that a substantial proportion of MDR-TB patients had annual household incomes less than 10,000 Yuan (≈1500 US dollars). It is considered to be a catastrophic payment by a household if a medical care charge is 40 % or more of the household’s capacity to pay [24]. In the field evaluation, we collected data on patient-reported treatment costs. However, we did not analyse these data because we could not determine direct and indirect medical costs and losses of income from the respondents’ reports. We strongly recommend that future studies conduct economic analyses because TB is a disease of poverty, and out-of-pocket payments could be an important predictor of treatment compliance and outcome.

This study has provided valuable information on the high burden of TB and MDR-TB in Henan Province, China. Unfortunately, we could not follow up all patients in the 30 counties included in the baseline survey in 2001 to investigate patients’ out of pocket cost for treatment. Efforts were made to select representative counties in the sample. A complete follow up study covering all 30 counties in the province is recommended to evaluate all surveyed patients in 2001 and determine fully long term outcomes. Some of the information collected in this study was based on self-report from respondents. Annual household income and socioeconomic status may have been underestimated. We designed our questionnaire and conducted field interviews to minimise reporting bias.

Our study has important implications for TB control in China and worldwide, especially for countries with high TB burdens [5, 25, 26]. The much longer treatment durations and the lower cure rates of MDR-TB, compared with non-MDR-TB, pose continuing threats to communities. Many recent studies indicate that drug-resistant TB is transmitted primarily to healthy people [27, 28] - despite a belief that drug resistant mycobacteria have lowered virulence and transmissibility. The high death rate of MDR-TB puts a huge economic, social, and psychological burden on patients, families, and communities.

In 2014, the 67^th^ World Health Assembly passed a resolution approving the new post-2015 Global TB Strategy, with its ambitious and unprecedented targets, and with its vision of ending TB as an epidemic disease by 2035. Our study has demonstrated that MDR is a strong predictor for mortality. Given this finding, as well as the longer treatment period needed (hence a prolonged period during which others in the community may be infected), prevention and control of MDR-TB should be the first priority in order to reach the targets.

Chinese TB control programs should target MDR-TB patients because they are at great risk for death and poor socioeconomic outcome. Treatment of drug-resistant TB is being implemented by the NTP in some project areas through the Global Fund to fight AIDS, Tuberculosis and Malaria (GF). Five cities in Henan Province were covered by GF projects including susceptibility testing and treatment for MDR-TB. A challenge to the province and to the whole country is that the GF stopped its support in China in June 2014. The Chinese administration has already started to expand access to MDR-TB care with diagnosis and treatment transferred to TB-designated hospitals from the Chinese Centres for Disease Control and Prevention. Clinicians who focus on individual cases need time to embrace public health concepts and increase their capacity for diagnosing and treating MDR-TB patients.

To alleviate the economic burden on TB patients, the Chinese government has designated MDR-TB as one of eight priority diseases eligible for 70 % reimbursement in the country’s rural health insurance program. However, the reimbursement proportion may need to be increased, and additional financial assistance may need to be provided to ensure that the poorest and most vulnerable patients have access to care and are able to complete their treatment.

The findings from this study highlight that MDR-TB is a serious public health problem in China. Global Fund support has permitted the scaling up of drug sensitivity testing and multi-drug resistant treatment in 921 counties of 92 prefectures in China, but sustainability after the Global Fund withdraws requires intensive Chinese government support.

## Conclusions

MDR-TB is a strong predictor for poor long-term outcome. High quality diagnosis and treatment must be ensured. Greater reimbursement or free treatment may be needed to provide access for the poor and vulnerable populations, and to increase treatment compliance.

## Footnotes

1 Treatment success was defined as either cure or treatment completed. Cure was defined as a patient whose sputum smear or culture was positive at the beginning of the treatment but smear- or culture-negative in the last month of treatment and on at least one prior occasion. Treatment completed did not need to meet the criteria for cure or failure. Failure was defined as sputum smear-positive at 5 months or later during treatment. Death was defined as a patient who died for any reason during the course of treatment (the cause of death during TB treatment was not recorded).
